# Health utilities and willingness to pay in adult patients with coeliac disease in Hungary

**DOI:** 10.1007/s11136-023-03418-w

**Published:** 2023-04-17

**Authors:** M. Mercédesz Angyal, Peter L. Lakatos, Balázs Jenei, Valentin Brodszky, Fanni Rencz

**Affiliations:** 1 Károly Rácz Doctoral School of Clinical Medicine, Semmelweis University, 26 Üllői út, Budapest, 1085 Hungary; 2 Department of Health Policy, Corvinus University of Budapest, 8 Fővám tér, Budapest, 1093 Hungary; 3 McGill University Health Centre, Montreal General Hospital, 1650 Ave. Cedar, D16.173.1, Montreal, QC H3G 1A4 Canada; 4 Department of Internal Medicine and Oncology, Semmelweis University, Korányi Sándor u. 2/a, Budapest, 1083 Hungary

**Keywords:** Coeliac disease, Gluten-free diet, Willingness to pay, Time trade-off, Health-related quality of life, Utility

## Abstract

**Background:**

Coeliac disease (CD) is a life-long food-related disorder with a global prevalence of approximately 1%. Patients with CD often experience reduced health-related quality of life that could improve with a strict adherence to a gluten-free diet (GFD).

**Objectives:**

To obtain visual analogue scale (VAS), time trade-off (TTO) and willingness-to-pay (WTP) values amongst patients with CD.

**Methods:**

In 2020–2021, a cross-sectional online survey was conducted amongst 312 adult CD patients in Hungary. Patients completed the Gastrointestinal Symptom Rating Scale (GSRS) and evaluated their current health and three hypothetical health state vignettes defined based on dietary adherence using VAS, conventional 10-year TTO and WTP. Multivariate regressions were used to explore the effect of patients’ demographic and clinical characteristics on utility and WTP values.

**Results:**

Mean VAS values for current health and ‘CD with strict adherence to GFD’, ‘CD with loose adherence to GFD’ and ‘CD without GFD’ hypothetical health states were 79.69 ± 18.52, 85.36 ± 16.18, 62.44 ± 19.91 and 36.69 ± 25.83, respectively. Corresponding mean TTO utilities were: 0.90 ± 0.19, 0.91 ± 0.20, 0.87 ± 0.23 and 0.76 ± 0.29. Mean annual WTP values for returning to full health were: €845 ± 1077, €648 ± 1002, €862 ± 1135 and €1251 ± 1496. Older age at diagnosis, male sex, more severe gastrointestinal symptoms (GSRS) and having comorbidities were associated with lower VAS and TTO or higher WTP values for current own health (*p* < 0.05).

**Conclusion:**

This is the first study to report TTO utilities for CD health states. Strict adherence to the GFD may result in substantial health gains in symptomatic patients. Utilities and WTP results can be used to estimate benefits of GFD in cost-utility and cost–benefit analyses.

## Introduction

Coeliac disease (CD) is an immune-mediated systemic disorder activated by the ingestion of gluten in genetically susceptible individuals [1]. It is the most common life-long food-related disorder with a global prevalence of 0.7–1.4% [2, 3]. CD can develop at any age and it is approximately 1.5 times more common in females than in males [2, 4, 5]. The clinical presentation is heterogeneous and may include gastrointestinal problems, signs of malabsorption, as well as extraintestinal symptoms, such as dermatitis herpetiformis, arthritis, neurological symptoms and anaemia [6]. CD is also associated with and increased risk of depression, anxiety, attention deficit hyperactivity disorder and eating disorders [7]. Currently, the only treatment option is a strict, life-long gluten-free diet (GFD) [8]. However, adherence to a GFD may be challenging in everyday life mostly due to the limited availability, high costs and variable quality of gluten-free products, and individual lifestyle aspects (difficulties with dining out and travelling as well as lack of social support) [9–12].

A large number of studies reported impaired health-related quality of life (HRQoL) in CD patients before diagnosis [13–16]. A recent meta-analysis concluded that dietary adherence significantly improves but does not normalise HRQoL in CD patients [17]. To date, HRQoL in adult CD patients has been assessed by using both generic (e.g. EQ-5D, SF-36, WHOQOL-BREF) and condition-specific questionnaires [e.g. Celiac Disease Questionnaire (CDQ), Coeliac Disease Quality of Life questionnaire (CDQL), Coeliac Disease Quality of Life measure (CD-QoL), Coeliac Disease Assessment Questionnaire (CDAQ)] [13, 18–22]. Only a subset of these HRQoL instruments allow to assign health utilities to different CD health states (i.e. preference-accompanied measures). Utilities reflect how preferred certain health outcomes are on a scale anchored on full health (1) and death (0) [23].

So far, the EQ-5D has been the most commonly used instrument to derive health utilities in CD patients [15, 16, 24–27]. However, its five dimensions (mobility, self-care, usual activities, pain/discomfort and anxiety/depression), and in particular the pain/discomfort dimension may be insensitive to symptoms, such as diarrhoea and constipation [28]. It is therefore possible that the EQ-5D overestimates the HRQoL of CD patients. This is supported by three earlier EQ-5D studies that reported similar or even higher utilities (representing a better HRQoL) in CD patients than amongst members of the general population [15, 16, 25]. Another limitation in previous studies is that the vast majority of diagnosed CD patient populations lived gluten free at the time of assessment, and thus, utilities prior to the start of GFD were only assessed retrospectively; however, retrospective assessment may be subject to recall bias [16, 23–25, 27, 29]. As a consequence, no reliable utility estimates are available for CD before treatment. Moreover, none of the existing studies focussed on the impact of different levels of dietary adherence on utilities.

When generic preference-accompanied measures are not feasible to assess utilities, vignette-based methods may be considered. A vignette is a description of a hypothetical health state that is directly valued by using a preference elicitation method [30]. As vignettes are hypothetical, they enable to elicit preferences for any health state. Preferences for the vignettes may be measured in several ways; for example, people may trade off life years (time trade-off, TTO) or risk of death (standard gamble) to improve health. TTO is the most widely used method to directly obtain health utilities [31]; nevertheless, no prior studies used this approach in CD patients. Furthermore, preferences may also be assessed by contingent evaluation, where respondents are asked to reveal the amount they would be willing to pay to improve their health. To date, two studies have employed this method in CD. One study measured WTP for CD screening in parents of children diagnosed with CD in Sweden, and another one surveyed adult patients with CD in Switzerland [32, 33]. However, no vignette-based WTP studies have been carried out in CD to date.

This study therefore aims to assess health states by visual analogue scale (VAS), TTO and WTP values in CD patients using a vignette-based study design.

## Methods

### Study design and population

An online cross-sectional survey was performed between November 2020 and January 2021 in Hungary. Permission for conducting the study was granted by the Research Ethics Committee of the Corvinus University of Budapest (reference no. KRH/390/2020). A convenience sample of CD patients were recruited through 30 different patient organisations and social media groups. Participation was voluntary and anonymous, no remuneration was provided. The survey was programmed in Qualtrics (Qualtrics 2020, Provo, UT, USA). To be included in the study, participants were required to be aged 18 years or over and to give their informed consent. All questions in the survey were mandatory with the exception of the income question, therefore respondents could not proceed to the next question without answering the previous one.

### Questionnaire

The questionnaire consisted of four parts. The first part included questions about CD-related clinical characteristics, including disease duration, comorbidities and adherence to GFD. This section was built on two earlier national surveys involving CD patients in Sweden and the UK [15, 16]. The second part consisted of different standardised questionnaires to assess symptoms, HRQoL and well-being. CD-specific symptoms were measured with Gastrointestinal Symptom Rating Scale (GSRS) [34]. The GSRS is widely used in CD patients, including clinical trials and it has showed good validity and responsiveness to both GFD and gluten challenge in this patient population, and additionally, it was already available in Hungarian-language at the time of the data collection [35–37]. Regulatory authorities, such as the FDA also have familiarity with the GSRS [38]. The GSRS evaluates common gastrointestinal symptoms experienced by patients, where 15 items combine into five domains: reflux (2 items), abdominal pain (3 items), indigestion (4 items), diarrhoea (3 items) and constipation (3 items). Each item has seven response options with descriptive anchors ranging from ‘no discomfort at all’ (= 1) to ‘very severe discomfort’ (= 7). The total score, calculated by adding up the item scores, may range from 15 to 105, where a higher score represents more health problems. In the third part of the survey, the patients were asked to assess their own health, followed by three hypothetical health state vignettes, which appeared in a randomised order. *Both* current own health and the hypothetical health state vignettes were valued by VAS and TTO and by contingent evaluation asking WTP. In the last part of the questionnaire, sociodemographic data, including age, sex, employment, place of residence, net household income and education, were collected.

### Development of coeliac disease health-state vignettes

The following three hypothetical health state vignettes were developed for this study: (1) CD without GFD, (2) CD with loose adherence to GFD and (3) CD with strict adherence to GFD. The vignettes were presented from a second-person perspective. The vignettes were primarily developed based on existing literature reviews [13, 17, 39–41]. A recently published model on concepts relevant when assessing health outcomes in CD summarises the signs and symptoms as well as broader HRQoL aspects in CD based on 28 original studies and stakeholder interviews with clinical experts and payers [41]. The model incorporates both gastrointestinal and non-gastrointestinal signs and symptoms of the disease alongside the following six HRQoL aspects: daily activities (e.g. negative impact on career or work), relationships (e.g. family life), social/leisure (e.g. dining out), sleep, dietary burden of GFD (e.g. difficulty adhering to GFD) and psychological impacts (e.g. anxiety or depression). These HRQoL impacts overlap with those covered by the most widely used patient-reported outcome measures in CD. Considering the conceptual model and a thorough review of the item content of CD-specific HRQoL measures and symptom scales, the following six areas were selected to be included in the health state vignettes based on judgement of a patient, a gastroenterologist professor and two health economists experienced in utility assessment: diet, gastrointestinal symptoms, work/school, physical activities, sleep/fatigue, mood and social life (Table 1). Comprehensibility of the descriptions was tested in an interview with a CD patient.

**Table 1:** Coeliac disease hypothetical health state descriptions (vignettes)

	Health state ‘A’: CD without GFD	Health state ‘B’: CD with loose adherence to GFD	Health state ‘C’: CD with strict adherence to GFD
Diet	You are not on a diet; you may eat all kinds of food you want. You do not have to check the ingredient lists of food products	You are on a special diet, which you more or less follow, so you often cannot eat all kinds of food you want. You need to check the ingredient lists of food products	You are on a special diet that you follow strictly, so you cannot eat all kinds of food you want. You need to check the ingredient lists of food products
Gastro-intestinal symptoms	After meals, you often experience bloating, constipation or diarrhoea on a weekly basis. Bloating is often associated with abdominal pain or cramps, nausea or vomiting	After some meals you may experience bloating, constipation or diarrhoea. Bloating is often associated with abdominal pain or constipation	After meals, you do not experience bloating, constipation or diarrhoea. You hardly ever have abdominal pain or cramps
Work/school	Your health makes it difficult to carry out your duties at work or school properly, you often have to take time off work or miss school for medical appointments. At work/school you can eat with your peers in the canteen	You are able to perform your work or school duties properly. You rarely have to take time off work or miss school for medical appointments. At work/school you cannot eat with your peers in the canteen	Your condition does not prevent you from carrying out your duties at work or school. At work/school, you cannot eat with your peers in the canteen
Physical activities	Your digestive complaints (bloating, diarrhoea, constipation, vomiting or abdominal pain) prevent you from exercising, doing chores or shopping. In general, you feel weak to perform physical activities	In rare cases, your digestive complaints (bloating, diarrhoea, constipation, vomiting or abdominal pain) may prevent you from exercising, doing chores or shopping. You sometimes feel weak to perform physical activities	You are not prevented from exercising, doing housework or shopping. You generally do not feel weak to perform physical activities
Sleep/fatigue	Your abdominal pain or cramps often prevent you from falling asleep. You are regularly tired and feeling low during the day, you find it difficult to concentrate and need more sleep at night	You sometimes experience tiredness and feel low during the day, but you have no difficulty with concentration. At night, you can sleep as much as you need	You are not tired or feel low during the day, and you do not have any difficulty with concentration. At night, you can sleep as much as you need
Mood and social life	You experience mood swings, you are periodically depressed, and you experience less desire for the company of others. You are able eat with your peers at any social event	Your mood is stable and you do not have depression. At social events, you are prevented from eating with your peers. You are often unable to eat at meetings because the café/restaurant cannot provide meals that suit your diet	Your mood is stable and you do not have depression. At social events, you are prevented from eating with your peers, and you have to plan meals in advance. You are often unable to eat at meetings because the café/restaurant cannot provide meals that suit your diet

### Health state valuation

#### Visual analogue scale (VAS)

For the hypothetical health states, a horizontal VAS was used with the endpoints of ‘the worst health you can imagine’ (= 0), and ‘the best health you can imagine’ (= 100). To measure patients’ own current health, we used the EQ VAS in an earlier section of the questionnaire. Note that the EQ-5D-5L including the descriptive system and EQ VAS were both completed by the patients; however, only the EQ VAS data were used for the present study [42]. The EQ VAS has identical endpoints to the VAS we used for the hypothetical health states, but it is vertically aligned.

#### Time trade-off (TTO)

The TTO method elicits utility values for imperfect health states by asking patients to make a trade-off between quality and length of life [23]. We opted to use a 10-year time frame, as this is the most commonly used duration in valuation studies in Hungary and beyond [43–51]. Patients were asked to imagine living in their current health or in a hypothetical CD-related health state for the next 10 years, followed by death. Then they had to indicate how many life years they would give up in order to regain full health. We used the top-down titration; thus, respondents were offered a predefined list with responses ranging from 10 to 0 years, with the smallest tradable amount of time being 6 months. TTO utilities were computed using the following formula:

$${\text{Utility}} = 1 - \frac{{{\text{Patient's}}\,{\text{answer}}}}{{10\,{\text{years}}}}$$

Suppose, for example, that a patient indicated to give up 2 years, yielding a *U* = (10–2)/10 = 0.8. There was no worse-than-dead task in this study, therefore utilities ranged from 0 (being dead) to 1 (full health).

#### Willingness to pay (WTP)

WTP measures the maximum amount of money an individual would be willing to pay to be free from their own symptoms or those described in the vignettes [52]. In our questionnaire, monthly WTP values were recorded in a closed question format with an open-ended ‘other’ response option. Sixteen predefined monthly amounts (in HUF) were offered to patients based on a previous survey: none; 500; 1,000; 2,000; 4,000; 6,000; 8,000; 10,000; 15,000; 20,000; 25,000; 30,000; 45,000; 60,000; 80,000 and 100,000 [53].

### Statistical analysis

To ensure homogeneity and high quality of data, respondents that (1) have not been diagnosed with CD by a physician or (2) filled in the questionnaire in less than eight minutes were excluded. Socio-demographic and clinical characteristics of the patients were analysed using descriptive statistics. The difference in GSRS scores between female and male patients was tested by Mann–Whitney *U* test. WTP responses were converted into a yearly value and then to euros, based on the European Central Bank’s closing conversion rate for February 2021 (EUR 1 = HUF 361.01). Nonsensical WTP responses (e.g. ‘I can not tell’) were excluded from the data analysis. Descriptive statistics (mean, median, standard deviation and IQR, proportion of ‘0’ and maximum responses) were computed for VAS, TTO and WTP values. The differences in VAS, TTO and WTP values across the patients’ own health and the three hypothetical health states were tested by Friedman test. Predictors of VAS, TTO and WTP values were explored by using multivariate regression models (OLS for own health and random-intercept linear models for hypothetical health states). Insignificant variables were removed from the models by backward stepwise elimination. Before the regressions, a logarithmic transformation was applied to normalise the distribution of the WTP responses. Heteroscedasticity was evaluated by Breusch–Pagan test and corrected by using robust standard errors. For all analyses, a *p* < 0.05 was considered statistically significant. Data were analysed using Stata 14.0 (StataCorp. 2015, College Station, TX, USA) and R 4.2.0 (R Core Team, 2022, Vienna, Austria).

## Results

### Sample characteristics

**Fig. 1:**
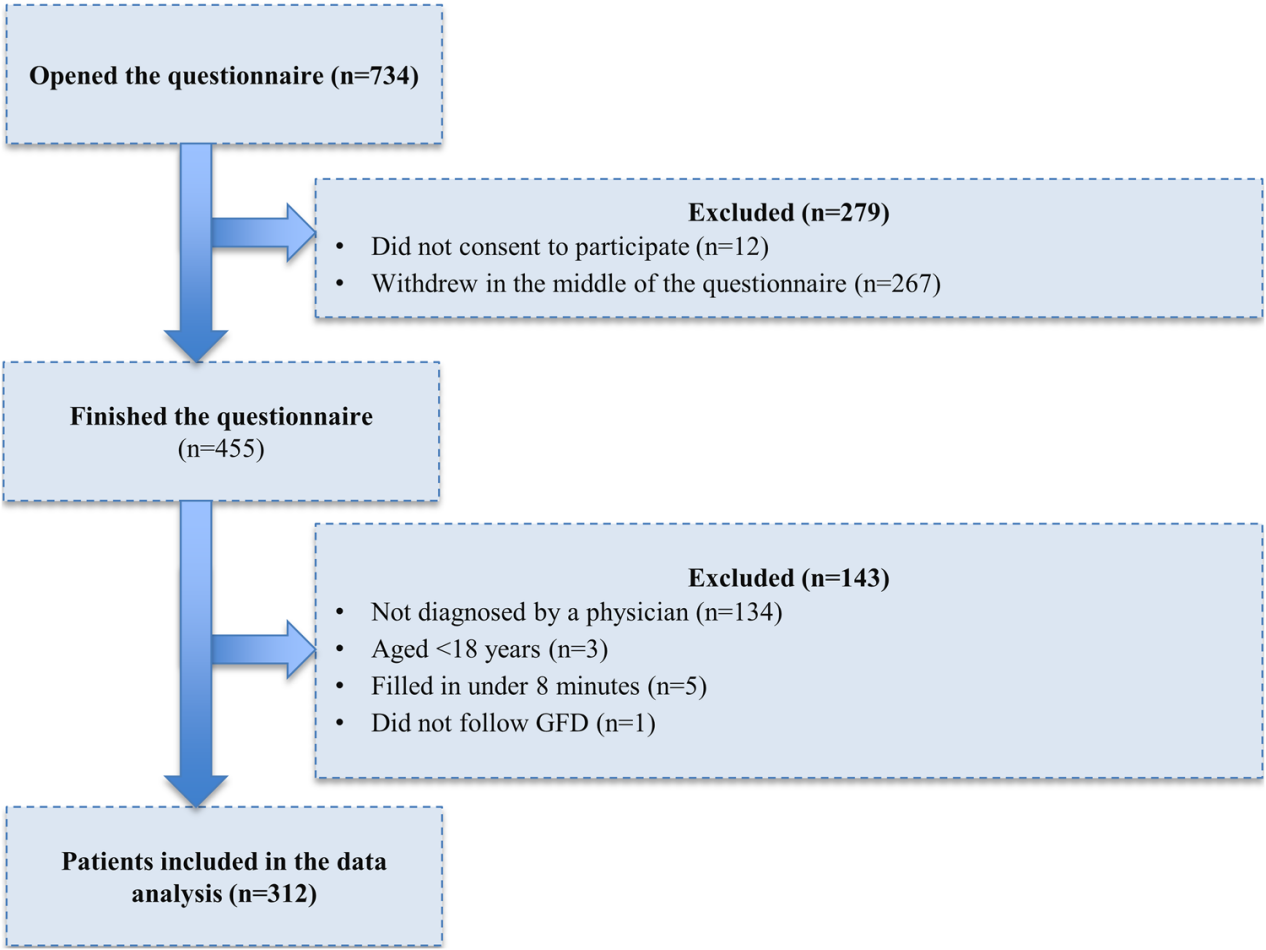
Study flow chart

Out of 734 individuals that opened the questionnaire, 455 (62.0%) finished it. Of these, 143 respondents were excluded based on the exclusion criteria (Fig. 1). Furthermore, one patient that reported not following GFD was decided to be excluded to ensure homogeneity of the sample. Thus, data of 312 physician-diagnosed CD patients were included in the analyses. Socio-demographic and clinical characteristics of patients are presented in Table 2. Mean age was 35.8 (SD = 11.5) ranging from 18 to 80 years. The majority of the patients were female (70.2%), and the average age at diagnosis was 27.1 years (SD = 14.0). The patient population was considerably younger and better educated than the adult general population in Hungary. Most patients worked in full-time positions or were self-employed (67.6%), and 29.8% of them lived in the capital. All patients followed GFD at the time of the survey. The majority (90.4%) of patients reported having comorbidities. The most common comorbidities were allergies (35.3%), other food intolerance (30.8%) and gastroesophageal reflux disease (27.6%). Overall, 23.7% of patients reported one, whilst 65.7% two or more comorbidities. Mean GSRS score was 28.3, and females reported significantly more problems than males (mean 29.62 vs. 25.02, *p* = 0.001).

**Table 2:** Characteristics of the study population

Variables	CD patient in the survey		General population reference value^a^
	Mean or *n*	SD or %	Mean or *n*
Sex			
Female	219	70.2	53.1
Male	93	29.8	46.9
Age (years)			
18–24	59	18.9	10.0
25–34	98	31.4	15.2
35–44	73	23.4	19.5
45–54	68	21.8	16.0
55+	14	4.5	39.3
Place of residence			
Capital	93	29.8	17.9
County town	69	22.1	52.6
Other town	76	24.4	
Village	74	23.7	29.5
Highest level of education			
Primary school	8	2.6	23.8
Secondary school	147	47.1	55.0
College/university	157	50.3	21.2
Employment			
Full-time/self employed	211	67.6	53.1
Part-time employed	19	6.1	
Student	44	14.1	4.7
Unemployed	10	3.2	3.1
Other (incl. retired)	28	9.0	30.2
Per capita net monthly household income (EUR)^b^	801.5	529.0	
1st quintile	266.4	87.3	n/a
2nd quintile	474.1	57.8	n/a
3rd quintile	649.0	48.6	n/a
4th quintile	954.9	133.8	n/a
5th quintile	1642.4	455.8	n/a
Don’t know/refused to answer	52	16.7	n/a
Following a gluten-free diet (GFD)	312	100	n/a
Age at diagnosis	27.1	14.0	n/a
Number of comorbidities			
0	33	10.6	n/a
1	74	23.7	n/a
2–3	101	32.4	n/a
4+	104	33.3	n/a
Most common comorbidities^c^			
Allergies	110	35.3	14.6
Other food intolerance	96	30.8	n/a
Gastroesophageal reflux disease	86	27.6	n/a
Hair loss	67	21.5	n/a
Thyroid disease	62	19.9	n/a
Iron deficiency	56	18.0	n/a
Eczema	44	14.1	n/a
Hypertension	40	12.8	30.9
Depression	37	11.9	8.0
Anaemia	34	10.9	n/a
Rheumatic disease	30	9.6	10.3
Inflammatory bowel disease	12	3.8	n/a
Gastrointestinal Symptom Scale (GSRS) score (total sample)	28.3	11.7	n/a
Female	29.6	12.0	n/a
Male	25.0	10.4	n/a

*n/a* not available or not applicable

^a^Hungarian Central Statistical Office: Microcensus 2016 and European Health Interview Survey in Hungary, 2019

^b^1 EUR equals to 361.01 HUF

^c^Multiple comorbidities could be selected

### VAS, TTO and WTP values

**Fig. 2:**
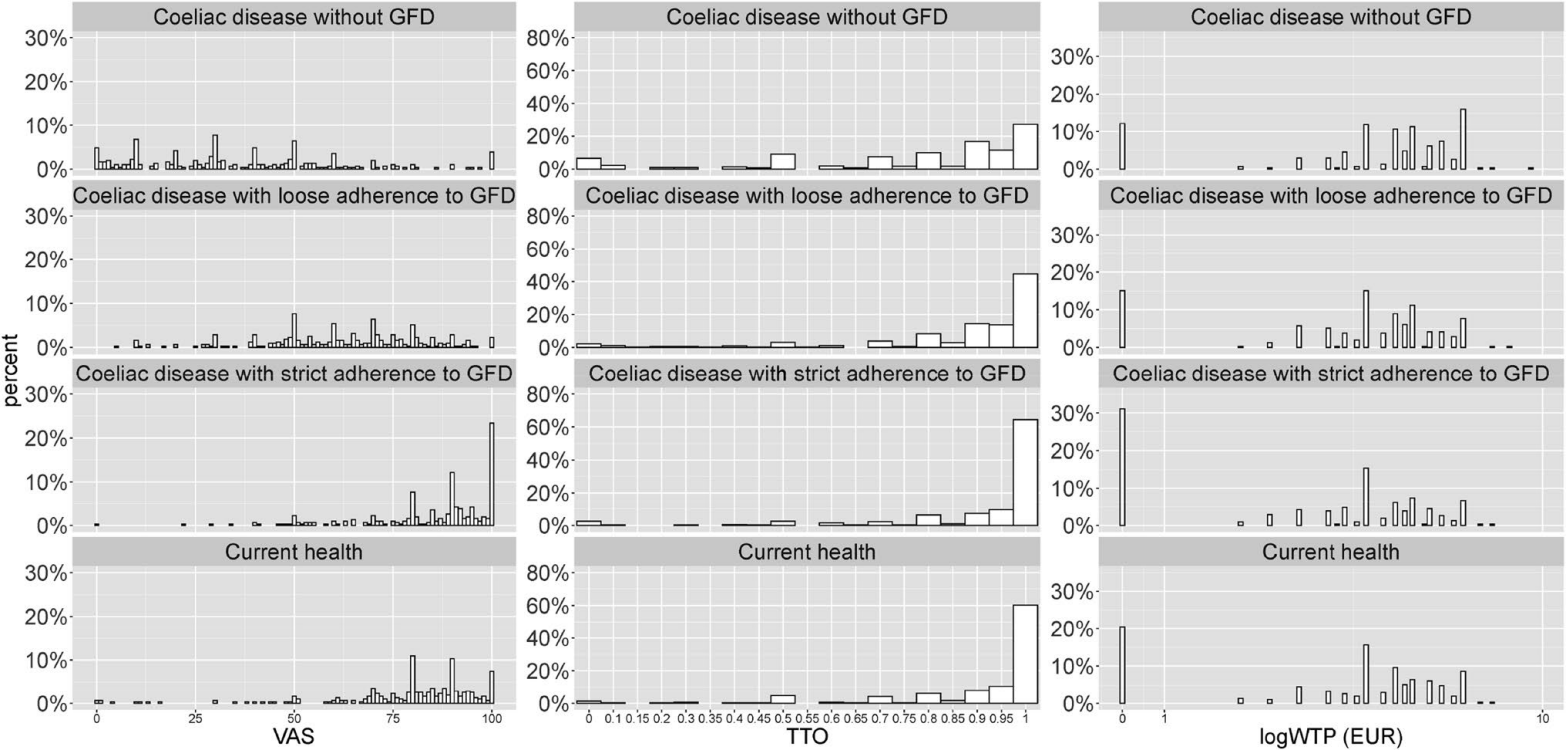
Distribution of VAS, TTO and WTP responses for four CD health states. *VAS* visual analogue scale, *TTO* time trade-off, *WTP* willingness to pay

The distribution of VAS, TTO and WTP values is depicted in Fig. 2. Mean VAS values for current health, ‘CD with strict adherence to GFD’, ‘CD with loose adherence to GFD’ and ‘CD without GFD’ hypothetical health states 79.69 ± 18.52, 85.36 ± 16.18, 62.44 ± 19.91 and 36.69 ± 25.83, respectively (Table 3). Corresponding mean TTO utilities were: 0.90 ± 0.19, 0.91 ± 0.20, 0.87 ± 0.23 and 0.76 ± 0.29. A total of 188 patients (60.3%) were not willing to give up any time for their current health, and there were 73 patients (23.4%) who refused to trade life years in any of the four TTO tasks (non-traders). Overall, 1.3%, 2.6%, 2.2% and 6.4% of the patients traded all the 10 years for the current health, ‘CD with strict adherence to GFD’, ‘CD with loose adherence to GFD’ and ‘CD without GFD’ hypothetical health states.

**Table 3:** Descriptive statistics of the VAS and TTO utilities and WTP values

Outcome	Health state	*n* (missing)	Mean	SD	Quartiles			‘0’ answers* (%)	Maximum answers** (%)
					Q1	Median	Q3		
VAS	Own health	312	79.69	18.52	75	83.50	90.00	0.6	7.4
	CD with strict adherence to GFD	312	85.36	16.18	80	90	98.75	0.3	23.4
	CD with loose adherence to GFD	312	62.44	19.91	50	64.5	77	0	2.2
	CD without GFD	312	36.69	25.83	15	31	50	4.8	3.9
TTO	Own health	312	0.90	0.19	0.9	1	1	1.3	60.3
	CD with strict adherence to GFD	312	0.91	0.20	0.9	1	1	2.6	64.4
	CD with loose adherence to GFD	312	0.87	0.23	0.85	0.95	1	2.2	44.6
	CD without GFD	312	0.76	0.29	0.70	0.90	1	6.4	27.2
WTP (EUR/year)	Own health	302 (10)	845	1077	66	332	997	20.5	9.3
	CD with strict adherence to GFD	311 (1)	648	1002	0	332	831	31.1	7.4
	CD with loose adherence to GFD	308 (4)	862	1135	133	499	997	15.1	8.3
	CD without GFD	305 (7)	1251	1496	299	831	1994	12.2	17.0

TTO time trade-off, VAS visual analogue scale, WTP willingness to pay

*Equal to 0 value in VAS, trading all the 10 years in TTO, €0 responses in WTP

**Equal to 100 value in VAS, trading 0 years in TTO, maximum annual amount in WTP (1 200 000 HUF—€3324 or above)

Currency change: EUR 1 = HUF 361.01

Of the 1248 WTP responses given in the four tasks, 22 (1.8%) nonsensical answers (from 13 patients) were excluded. The mean annual WTP values were €845 ± 1077 for current health, €648 ± 1002 for the ‘CD with strict adherence to GFD’ health state, €862 ± 1135 for the ‘CD with loose adherence to GFD’ health state and €1251 ± 1496 for ‘CD without GFD’ health state. Overall, 79.5%, 68.9%, 84.9% and 87.8% were willing to pay to regain full health. The maximum WTP was €16,620 annually (‘CD without GFD’ health state). A total of 20 patients (6.4%) refused to pay for any of the health states, including their own health. Using any of the three methods (VAS, TTO and WTP), there was a statistically significant difference across patients’ valuations for the four health states (*p* < 0.001).

### Predictors of current own health VAS, TTO and WTP values

The multivariate regressions showed that patients with higher income had significantly higher VAS values (p = 0.006) (Table 4). A one-point increase in GSRS score (indicating worse HRQoL) was associated with an average decrease of 0.421 (*p* < 0.001), and a 1-year older age at diagnosis with a 0.173 decrease in VAS values (*p* < 0.05). Concomitant depression and rheumatic disease substantially reduced patients’ VAS values (− 10.373 and − 12.197 *p* < 0.05). These variables together explained 30.9% of the overall variation in VAS values.

**Table 4:** Predictors of the VAS and TTO utilities

VAS	Coefficient (ß)	SE	*p* value
Current health (dependent variable: EQ VAS values): linear regression, *n* = 260 *R*^2^ = 0.309			
Constant	62.492	13.848	< 0.001
Household’s per capita net annual income (EUR, logarithm)	3.986	1.431	0.006
GSRS score	− 0.421	0.119	< 0.001
Age at diagnosis (years)	− 0.173	0.077	0.026
Comorbidity: depression	− 10.373	4.593	0.025
Comorbidity: rheumatic disease	− 12.197	5.170	0.019
Hypothetical health states (dependent variable VAS): random-intercept regression, *n* = 312, *R*^2^ = 0.490			
Constant	91.015	2.240	< 0.001
Hypothetical health states			
‘CD without GFD’ health state	− 48.673	1.530	< 0.001
‘CD with loose adherence to GFD’ health state	− 22.917	1.530	< 0.001
Individual characteristics			
GSRS score	− 0.168	0.070	0.016
Comorbidity: rheumatic disease	− 5.872	2.688	0.029
Comorbidity: inflammatory bowel disease	− 8.763	4.200	0.037

TTO	Coefficient (ß)	SE	*p* value
Current health (dependent variable: years of TTO): linear regression, *n* = 260 *R*^2^ = 0.0856			
Constant	0.609	0.198	0.002
Female	0.068	0.024	0.005
Household’s per capita net annual income (EUR, logarithm)	0.039	0.020	0.055
GSRS score	− 0.004	0.001	0.005
Hypothetical health states (dependent variable TTO): random-intercept regression, *n* = 260, *R*^2^ = 0.0873			
Constant	0.380	0.167	0.024
Hypothetical health states			
‘CD without GFD’ health state	− 0.145	0.015	< 0.001
‘CD with loose adherence to GFD’ health state	− 0.038	0.015	0.011
Individual characteristics			
Household’s per capita net annual income (EUR, logarithm)	0.050	0.018	0.004
Age (years)	0.002	0.001	0.045

*GSRS* gastrointestinal symptom rating scale, *TTO* time trade-off, *VAS* visual analogue scale, *WTP* willingness to pay

Females and those with higher income were, on average, willing to trade less life years, resulting higher TTO utilities (*p* < 0.05) (Table 4). Similarly to VAS, a one-point increase in GSRS resulted in a 0.004 decrease in TTO utilities (*p* = 0.005). These three variables explained 8.6% of the variance of TTO utilities.

The amount patients were willing to pay decreased by 70.45% in females compared to males (*p* = 0.022). Patients’ WTP increased by 6.8% with + 1 GSRS score (*p* = 0.002), by 3.9% with a 1-year increase in age at diagnosis (*p* = 0.047) and by 251.6% in case of concomitant gastroesophageal reflux disease (*p* = 0.012) (Table 5). A 1% increase in the household’s per capita net annual income was associated with an 1.33% increase in the willingness-to-pay amount, on average (*p* < 0.001).

**Table 5:** Predictors of WTP responses

	Coefficient (ß)	SE	*p* value	% Change effect
Current health (dependent variable: logarithm of WTP): log-linear model, *n* = 253 *R*^2^ = 0.145				
Constant	− 9.887	3.460	0.005	–
Individual characteristics				
Female	− 1.219	0.527	0.022	− 70.454
Household’s per capita net annual income (EUR, logarithm)	1.331	0.352	< 0.001	–
GSRS score	0.066	0.021	0.002	6.842
Age at diagnosis (years)	0.038	0.019	0.047	3.894
Comorbidity: gastroesophageal reflux disease	1.257	0.498	0.012	251.614
Hypothetical health states (dependent variable logarithm of WTP): random-intercept log-linear model, *n* = 259, *R*^2^ = 0.109				
Constant	− 10.400	2.765	< 0.001	–
Hypothetical health states				
‘CD without GFD’ health state	2.020	0.230	< 0.001	653.811
‘CD with loose adherence to GFD’ health state	1.482	0.229	< 0.001	340.335
Individual characteristics				
Household per capita net annual income (EUR, logarithm)	1.393	0.291	< 0.001	–
GSRS score	0.048	0.017	0.005	4.937

*GSRS* gastrointestinal symptom rating scale, *TTO* time trade-off, *WTP* willingness to pay

### Predictors of VAS, TTO and WTP values for hypothetical CD health states

Both the ‘CD without GFD’ and the ‘CD with loose adherence to GFD’ hypothetical health states were associated with significantly lower VAS and TTO and higher WTP valuations compared to the ‘CD with strict adherence to GFD’ health state (*p* < 0.05) (Tables 4, 5). A + 1 GSRS score, indicating worse HRQoL was associated with an, on average, 0.168-point decrease in the VAS values and with a 4.9% increase in the WTP amount (*p* < 0.05). Having rheumatic or inflammatory bowel disease decreased the patients’ VAS valuations and having higher income resulted in higher TTO and WTP values (*p* < 0.05). Furthermore, every 1-year increase in the patients’ age increased the TTO utility by 0.002 (*p* = 0.045). These variables explained 49.0%, 8.7% and 10.9% of the overall variation in VAS, TTO and WTP values, respectively.

## Discussion

This study aimed to provide VAS, TTO and WTP values in adult patients with CD. To our knowledge, this is the first study to report TTO utilities in CD patients. The health state ‘CD with strict adherence to GFD’ had lower utilities than expected, possibly reflecting the psychosocial aspects of the disease, for example, the patients being prevented from eating with their peers. Older age at diagnosis, male sex, more severe gastrointestinal symptoms and having comorbidities were associated with lower VAS and TTO or higher WTP values for current own health. Compared to the TTO and WTP values, a relatively higher proportion of the variance of VAS values was explained by sociodemographic and clinical predictors. A possible explanation for this observation is that our study did not collect data on some likely predictors of TTO, including cultural values, self-esteem, marital status, having children, religious beliefs and attitudes towards life [54–56]. Similarly WTP values may have various additional predictors, such as sociodemographic characteristics and perceived threats and benefits of treatment [57].

It is worthwhile to reconcile our findings with those of previous studies with CD patients. The mean GSRS score in our study (28.3) was in the range of other patient populations on GFD from different countries (21.0–30.4) [58, 59]. Females reported significantly more problems on GSRS compared to males, which is in line with the existing literature suggesting that women with CD experience a greater deterioration in their HRQoL than men [60–64]. Despite the higher average GSRS score in our study, women were willing to trade less life years in the TTO and pay less in order to regain full health. Older age at diagnosis was associated with lower VAS and higher WTP values, similarly the literature, which suggests that a late diagnosis of CD can lead to a higher morbidity and lower HRQoL [59, 65–69]. The current health VAS values of the Hungarian CD patients on GFD (79.69) are in accordance with those reported in previous cross-sectional studies from Poland (75.1) with 93.7% of the patients following the GFD all the time, and the UK (80.00) with 90.8% of the patients following the GFD all the time [15, 26, 27]. Mean EQ-5D-3L utilities in CD patients before the diagnosis (assessed retrospectively) were 0.56 and 0.65 in two UK in 2006 and 2015, whilst after diagnosis these improved to 0.84 and 0.85 [15, 27]. Similarly, in Poland, the pre- (assessed retrospectively) and post-diagnosis mean EQ-5D-5L utilities were 0.79 and 0.94 [26]. These results are comparable to our findings, whereby the hypothetical health state of ‘CD without GFD’ had a mean TTO utility of 0.76 and the ‘CD with strict adherence to GFD’ health state had a mean TTO utility of 0.91.

Preference measurement also helps to shed light on the HRQoL burden associated with a disease. In addition, utilities may be used to calculate quality-adjusted life years (QALYs) in cost-utility analyses, whilst WTP values may be used in cost–benefit analyses of GFD and possible new treatments in the future [70]. Preferences may be derived from the general public or patients. In most European countries, a societal perspective is recommended in the context of economic evaluations in healthcare [71–73]. However, there is a growing amount of literature arguing that utilities based on both patient and general population preferences ought to be considered in economic analyses [74–76].

CD without a strict adherence to GFD might result in a sizeable QALY loss at a societal level. Preference-accompanied measures, such as the EQ-5D, may not be able to fully capture the health impact of CD [28], therefore vignette-based methods might be superior to indirect utility assessment in this patient population. We found that the TTO method discriminated well between health states according to dietary adherence. In cost-utility analyses of GFD and new treatments for CD, directly elicited utilities may be recommended to be used to calculate QALYs. Over 10 years, untreated CD may cause a loss of between 1.3 (with loose adherence to GFD, calculated as 10*(1–0.87)) and 2.4 (CD without GFD, calculated as 10*(1–0.76)) QALY per patient. These findings highlight the large health gains associated with GFD and may be considered when quantifying effectiveness of programmes to support CD patients’ access to gluten-free food products. The WTP results also provide insights for the industry to invest in research and development in alternative treatment methods for CD, given that 69–88% of patients were willing to pay in our WTP tasks. Furthermore, the WTP data from this study will be useful for cost–benefit analyses of GFD.

Many countries offer various forms of reimbursement for CD patients, such as tax reduction (Hungary, Canada, the US, the Netherlands and Portugal), cash transfer (Italy, Argentina, Uruguay, Finland, Greece, France, Norway, Belgium and Slovenia only for children), food provision (some provinces in Argentina and Spain), prescription for gluten-free food (New Zealand, Ireland, the UK) and subsidy (Northern Ireland, Scotland, the Czech Republic) to reduce the individual financial burden of GFD [77, 78]. However, other countries or regions provide no coverage of GFD products at all (e.g. Germany, some provinces in Spain, Mexico) [78]. Our findings may contribute new evidence for relevant national health and social policy programmes affecting the access to gluten-free products.

This study has a few limitations that should be noted. First, the questionnaire was administered online and relied on self-reported clinical data that were not verified by physicians. Secondly, selection bias might have occurred as the majority of our study population were from the middle- and high-income social groups, with college or university degrees, and females were somewhat overrepresented in the study population. It is also possible that patients voluntarily filling in such a questionnaire may differ in their clinical characteristics, and thus, the sample may not be representative of the whole population of CD patients in Hungary. In the valuation tasks, three different hypothetical health states were valued; however, the clinical manifestation may vary widely and other CD health states with atypical symptoms could have also been selected. Given that the majority of patients respond to GFD, we only included hypothetical health states that improve after following the GFD. However, a substantial minority may develop persistent or recurrent symptoms even after following GFD [1]. Furthermore, only symptomatic hypothetical health states were considered and there were no health states describing silent celiac disease due to the limited evidence of the natural history thereof [79]. Albeit, several studies found that up to 15% of patients with positive serologic test (without histological confirmation) develop symptoms after 10 to 45 years [80–82]. An additional limitation of the study is that we did not decompose the overall higher utility for the GFD into its components, i.e. the possible utility loss associated with following the GFD (e.g. difficulties of keeping the diet) and the utility gain as a result of the improved HRQoL due to the diet. Finally, TTO utilities in this study may be somewhat upward biased due to the top-down titration approach, and we used the EQ VAS to assess VAS values for current health, but a horizontal VAS for the three hypothetical states that might not be entirely equivalent [83].

To conclude, this study provides a better understanding of the burden of CD by reporting VAS, TTO and WTP values for patients’ current health and three hypothetical GFD-related health states. Utilities and WTP results from the present study may be useful in economic evaluations examining and comparing the value of GFD, screening strategies, subsidies and new alternative treatment options in the future.

## Data Availability

All data of this study are available from the corresponding author upon reasonable request.

## Code availability

N/A.
